# Supplementary material for: Organ-specific immune responses are strain-dependent in a mouse model of Cryptococcus neoformans brain infection
Source: Microbiol Spectr. 2026 Feb 2;14(3):e02517-25. doi: 10.1128/spectrum.02517-25 (PMC12955457; doi:10.1128/spectrum.02517-25)
Supplement: Supplemental material — Fig. S1 and S2. [file spectrum.02517-25-s0001.pdf]

Supplementary Figures

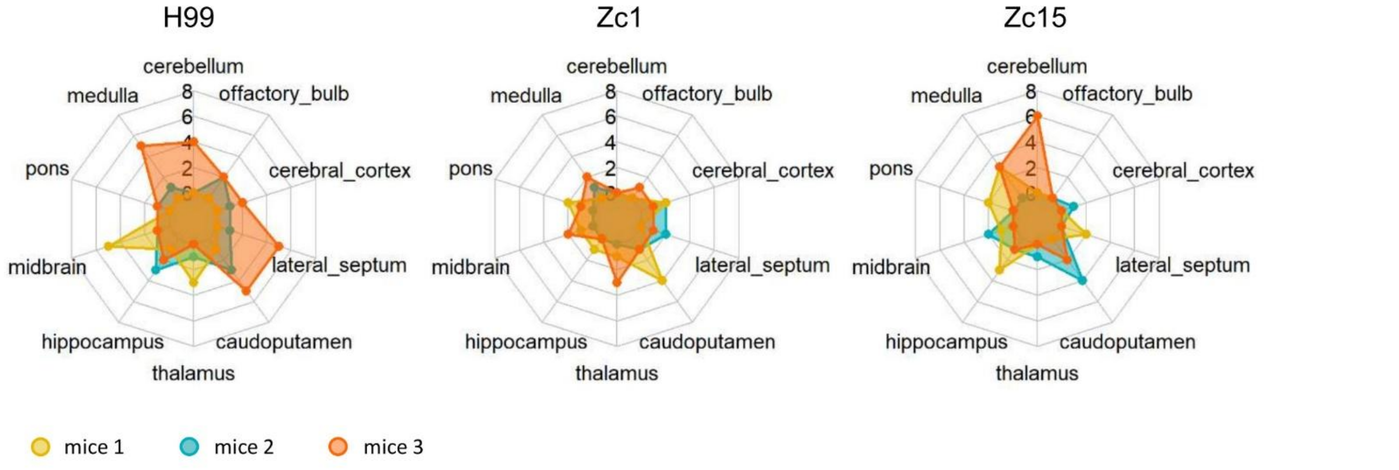

Figure S1: Number of lesions in different brain regions of mice infected with *C. neoformans* H99, Zc1 and Zc15. Histology was performed 7 days post-infection, stained with PAS. Three mice per fungal strain were analyzed.

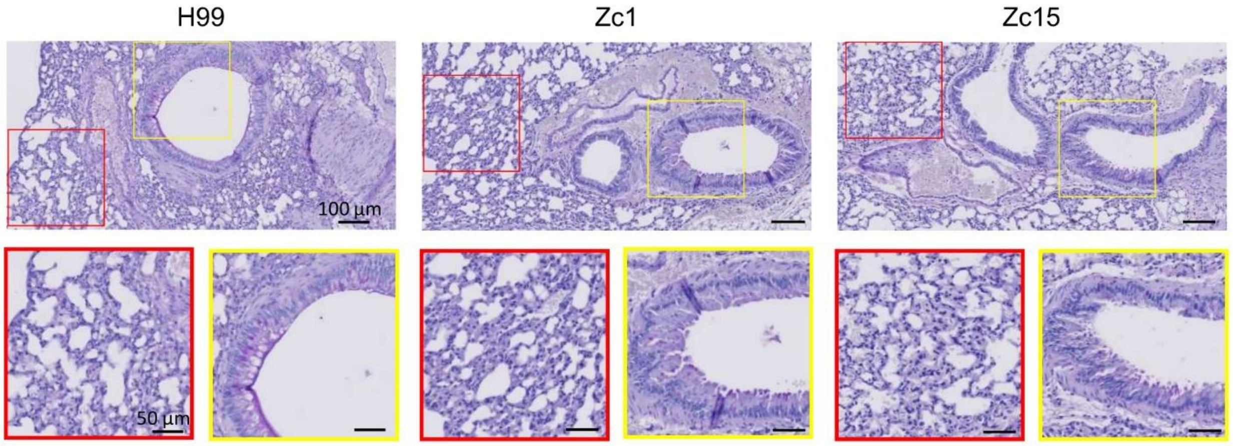

Figure S2: Representative histology from lung of mouse infected with *C. neoformans* H99, Zc1 and Zc15. Histology was performed 7 days post-infection, stained with PAS. Although cryptococcal cells are absent in these regions, alveolar thickening (red boxes), increasing bronchial mucosal hyperplasia and hypertrophy (yellow boxes) and PAS-positive stained cells in bronchioles (yellow boxes) are present.
